# Crystal structure of the flavoenzyme PA4991 from *Pseudomonas aeruginosa*

**DOI:** 10.1107/S2053230X15024437

**Published:** 2016-01-22

**Authors:** Agata Jacewicz, Robert Schnell, Ylva Lindqvist, Gunter Schneider

**Affiliations:** a Department of Medical Biochemistry and Biophysics, Karolinska Institutet, S-171 77 Stockholm, Sweden

**Keywords:** molecular replacement, *Rosetta* model, phasing, oxidoreductase, FAD

## Abstract

The locus PA4991 in *Pseudomonas aeruginosa* encodes an open reading frame that has been identified as essential for the virulence and/or survival of this pathogenic organism in the infected host. Here, it is shown that this gene encodes a monomeric FAD-binding protein of molecular mass 42.2 kDa. The structure of PA4991 was determined by a combination of molecular replacement using a search model generated with *Rosetta* and phase improvement by a low-occupancy heavy-metal derivative. PA4991 belongs to the GR_2_ family of FAD-dependent oxidoreductases, comprising an FAD-binding domain typical of the glutathione reductase family and a second domain dominated by an eight-stranded mixed β-sheet. Most of the protein–FAD interactions are *via* the FAD-binding domain, but the isoalloxazine ring is located at the domain interface and interacts with residues from both domains. A comparison with the structurally related glycine oxidase and glycerol-3-phosphate dehydrogenase shows that in spite of very low amino-acid sequence identity (<18%) several active-site residues involved in substrate binding in these enzymes are conserved in PA4991. However, enzymatic assays show that PA4991 does not display amino-acid oxidase or glycerol-3-phosphate dehydrogenase activities, suggesting that it requires different substrates for activity.

## Abstract 2

PA4991 is a FAD-dependent oxidoreductase from the pathogen *P. aeruginosa* that is essential for virulence and survival in the infected host. The structure of this enzyme, determined to 2.4 Å resolution, reveals that PA4991 belongs to the GR_2_ family of flavoenzymes.

## 1. Introduction   

Infections by Gram-negative bacteria are particularly difficult to treat owing to the limited repertoire of antibiotics against these pathogens, increasing drug resistance and their tendency to form persistent infections (Zavascki *et al.*, 2010 ▸). The ubiquitous pathogen *Pseudomonas aeruginosa* is able to adapt to a variety of environmental conditions and is therefore associated with a broad range of pathologies, including respiratory-tract, blood and skin infections. *P. aeruginosa* infections are mainly found in patients with immunosuppression, burns or cystic fibrosis (Kerr & Snelling, 2009 ▸). Increasing challenges in the clinical treatment of these infections underline the need to identify novel targets and lead compounds for the development of antibacterial agents, in particular those that target Gram-negative pathogens (Rice, 2008 ▸).

High-throughput genomic approaches have been employed to identify genes essential for the virulence and/or survival of pathogenic organisms in the infected host. Randomized transposon mutagenesis screens using *P. aeruginosa* strains PAO1 and P14 provided an estimate of 300–400 essential genes in PAO1 (Jacobs *et al.*, 2003 ▸) and 335 genes in P14 (Liberati *et al.*, 2006 ▸). Amongst these genes are PA4991 and PA4992, which are located on the same operon. A recent study using a transposon-sequencing and Monte Carlo simulation-based approach provided compelling evidence that the two genes are essential for growth of *P. aeruginosa* strains PAO1 and P14 in the sputum of cystic fibrosis patients (Turner *et al.*, 2015 ▸).

The proteins encoded by these two open reading frames were included in a larger study aimed at the characterization and assessment of potential targets in *P. aeruginosa* for early drug discovery (Moynie *et al.*, 2013 ▸). PA4992 belongs to the family of aldo–keto reductases, folding into an (β/α)_8_ barrel. NADPH binds at the C-terminal end of the β-strands, close to helix α8. PA4992 also contains a catalytic triad, Asp66, Tyr71 and Lys94, typical of this enzyme family (Moynie *et al.*, 2013 ▸).

Here, we report the crystallographic structure analysis of PA4991. The structure was determined by a combination of molecular replacement using a search model generated with *Rosetta* (Kim *et al.*, 2004 ▸) and phase improvement employing a low-occupancy heavy-metal derivative. We show that the enzyme belongs to a family of FAD-dependent oxido­reductases and compare its three-dimensional structure with those of other members of this family. Enzymatic assays suggest that PA4991 has a different substrate spectrum to its closest relatives glycerol-3-phosphate dehydrogenase and glycine oxidase.

## 2. Materials and methods   

### 2.1. Cloning, gene expression and protein purification   

The coding sequence of PA4991 was amplified by PCR from *P. aeruginosa* PAO1 genomic DNA using appropriate amplification primers adapted to the ligation-independent cloning method and cloned into pNIC28BSA4 (GenBank Accession No. EF198106) as described previously (Moynie *et al.*, 2013 ▸). The recombinant protein carries a TEV-cleavable His_6_ tag at the N-terminus. For a typical protein preparation, *Escherichia coli* BL21(DE3) cells harbouring the expression construct were grown at 37°C in 3 l LB medium supplemented with kanamycin (30 mg l^−1^) until the OD_600_ value reached 0.3, followed by growth for 1 h at 20°C, after which expression was induced by the addition of 0.2 m*M* IPTG. The culture was grown under inductive conditions at 20°C for 24 h.

After harvesting by centrifugation at 4000*g* and 4°C, the *E. coli* cells were resuspended in lysis buffer consisting of 20 m*M* Tris–HCl, 0.2 *M* NaCl, 10 m*M* imidazole pH 8.0. Lysozyme and DNAse were added to concentrations of 0.04 and 0.004 mg ml^−1^, respectively, and the lysis mixture was incubated at 22°C for 1 h. The cells were subsequently disrupted by sonication on ice and the lysate was clarified by centrifugation (25 000*g*) at 4°C. The supernatant was loaded onto 1.5 ml Ni^2+^ resin (Qiagen) equilibrated with lysis buffer and the column was washed with 50 ml of a solution consisting of 20 m*M* Tris–HCl, 0.2 *M* NaCl, 10 m*M* imidazole pH 8.0. The protein was eluted with a 10–500 m*M* imidazole gradient in 20 m*M* Tris–HCl buffer pH 8.0 containing 0.2 *M* NaCl. PA4991-containing fractions were combined and 0.7 mg TEV protease was added to the sample and incubated overnight at room temperature. After TEV cleavage, the sample was diluted five times with 40 m*M* Tris–HCl buffer pH 8.5 and concentrated to a total volume of 2 ml. The sample was loaded onto a 5 ml HiTrap Q HP column (GE Healthcare) equilibrated with 4% 40 m*M* Tris–HCl buffer containing 1 *M* NaCl. The recombinant protein was eluted with a 4–100% gradient of this buffer. The fractions containing PA4991 were collected and the sample was concentrated to a volume of 2 ml using a Vivaspin (Sartorius, Göttingen, Germany) centrifugation concentrator device with a 10 kDa molecular-weight cutoff. The sample was then loaded onto a Superdex 200 column (GE Healthcare, Uppsala, Sweden) with running buffer consisting of 10 m*M* Tris–HCl pH 8.0, 80 m*M* NaCl. The fractions containing pure (>95%) PA4991 were collected and concentrated to 40 mg ml^−1^ and aliquots were flash-frozen in liquid nitrogen. Selenomethionine-substituted PA4991 was produced according to the metabolic inhibition method (Van Duyne *et al.*, 1993 ▸) and was purified as described above.

### 2.2. Enzyme assays   

Enzymatic assays using recombinant PA4991 were carried out to probe for glycerol-3-phosphate dehydrogenase activity spectrophotometrically at 340 nm using both possible co-substrates: NAD^+^ and NADP^+^. The reaction mixtures consisted of 50 m*M* Tris–HCl, 100 m*M* NaCl pH 7.0, 1 m*M* glycerol-3-phosphate, 0.5 m*M* NAD^+^ or NADP^+^ and PA4991 at 1 µ*M* concentration. Similarly, the reverse reaction based on the reduction of dihydroxyacetone-3-phosphate with NADH or NADPH was assayed.

FAD-dependent amino-acid oxidase activity, which results in the release of H_2_O_2_, was assayed using a colorimetric method described previously (Su *et al.*, 2011 ▸). All 20 proteinogenic amino acids, the d-amino acids d-Ala and d-Glu, several nonstandard amino acids (l-2-aminopimelate, d-2-aminopimelate, *meso*-diaminopimelic acid, α-amino-adipate, l-norleucine, *S*-carboxy-l-cysteine, *S*-aminoethyl-l-cysteine, *N*-acetyl-l-cysteine, *O*-acetylserine and Gly-Gly) and nine amines (6-aminohexanoic acid, 1,5-diaminopentane, 1,6-diaminohexane, 1,8-diaminooctane, taurine, betaine, spermidine, spermine and sarcosine) were tested as potential substrates. The reaction mixtures consisted of 50 m*M* Tris–HCl, 100 m*M* NaCl pH 7.0, 2 m*M* of the putative substrate and PA4991 at 0.5 µ*M* concentration. The detection limits for both assays were in the range 1–5 turnovers per minute.

### 2.3. Crystallization and data collection   

Crystallization screens (JCSG+ from Qiagen and Wizard I+II from Emerald Bio) were carried out with native and selenomethionine-substituted PA4991 at protein concentrations ranging from 11.3 to 19.3 mg ml^−1^ using a Phoenix crystallization robot. Droplets of 100 nl protein solution (10 m*M* Tris–HCl pH 8.0, 80 m*M* NaCl) were mixed with the same amount of mother liquor and left to equilibrate at 4 or 20°C. One hit for native PA4991 could be reproduced in 24-well format, resulting in crystals that were suitable for data collection. Droplets of 1 µl protein solution were mixed with 1 µl mother liquor (0.2 *M* trimethylamine *N*-oxide, 20% PEG 2000 monomethyl ether, 0.1 *M* Tris–HCl pH 8.5) and equilibrated against 1.0 ml mother liquor at 20°C.

For the preparation of heavy-metal derivatives, crystals were soaked overnight in solutions of various salts [Pt(NH_3_)_2_Cl_2_, HgCl_2_ and phenylmercuric chloride] dissolved at concentrations of 1 and 2 m*M* in 0.2 *M* trimethylamine *N*-oxide, 20% PEG 2000 monomethyl ether, 0.1 *M* Tris–HCl pH 8.5.

Before data collection, crystals were transferred to a solution consisting of 30% PEG 2000 monomethyl ether, 0.2 *M* trimethyl­amine *N*-oxide, 80 m*M* NaCl, 10 m*M* Tris–HCl pH 8.0, 5%(*w*/*v*) glycerol and flash-cooled in liquid nitrogen. The X-ray diffraction data were collected at 100 K on beamline ID14-4 at ESRF, Grenoble, France. The data sets were integrated with *MOSFLM* (Leslie, 2006 ▸) and scaled using *SCALA* from the *CCP*4 suite (Winn *et al.*, 2011 ▸). The best diffracting crystal (2.4 Å resolution) was obtained by soaking native PA4991 crystals in 1 m*M* phenylmercuric chloride overnight and these data were used in the crystallographic analysis (Table 1 ▸).

### 2.4. Structure determination   

Initial attempts to determine the structure of PA4991 using anomalous data collected from crystals of the phenylmercuric chloride derivative were not successful. The program *CRANK*2 (Skubák & Pannu, 2013 ▸) located and refined two metal sites, but the resulting electron-density map was un­interpretable and the sites were later shown to be false. The structure was therefore determined by molecular replacement with *Phaser-MR* (Bunkóczi *et al.*, 2013 ▸). Initially, coordinates of putative homologues with PDB codes 2q6u (Carrell *et al.*, 2007 ▸) and 2r4j (Yeh *et al.*, 2008 ▸), both displaying less than 18% sequence identity to PA4991, were obtained from the PDB. Search models were derived from these coordinates by removing side chains and loop regions differing between the two structures, but no molecular-replacement solutions were found. The amino-acid sequence of PA4991 was then submitted to the *Robetta* server (http://robetta.bakerlab.org/) that runs the *Rosetta* software (Kim *et al.*, 2004 ▸), resulting in a best model with a score of 0.51. The five best models from *Robetta* were superimposed and all residues that showed significant deviations between these models were removed. The truncated models were used individually or as a search ensemble in molecular replacement, but none of these protocols yielded a clear solution. A second round of model trimming left only those parts of the best model that had the highest confidence as judged by *Robetta*, *i.e.* three strands and two helices of the FAD-binding domain, as a search model (*i.e.* 60 out of 391 amino-acid residues). Molecular replacement using this truncated model finally gave one solution that was clearly above the background, but with very low scores: RFZ = 4.9, TFZ = 4.8, PAK = 0, LLG = 30. From the phases obtained some more residues could be built; however, we did not deem it possible to bootstrap the complete structure. We then used *Phaser-EP* (McCoy *et al.*, 2007 ▸) and the obtained molecular-replacement model to locate and refine Hg sites and carry out phase improvement with *Parrot* (Zhang *et al.*, 1997 ▸). This procedure was repeated interspersed with the *Buccaneer* pipeline (Cowtan, 2006 ▸) and manual model building with *Coot* (Emsley *et al.*, 2010 ▸) until a complete structural model of PA4991 was obtained and refined. *Phaser-EP* was able to localize two correct Hg sites (included in the final model with occupancy 0.35), but also included some false sites during this procedure. During the model-building procedure electron density appeared for the cofactor FAD, which had not been included in any of the search models, thus indicating that the structure solution was correct. The final refinement run with *REFMAC*5 (Winn *et al.*, 2001 ▸) included TLS refinement.

*MolProbity* (Chen *et al.*, 2010 ▸) was used for validation. The final model contains one polypeptide chain of PA4991, one FAD molecule, two Hg^2+^ ions and 41 water molecules. The crystallographic data for PA4991 have been deposited in the Protein Data Bank (PDB entry 5ez7). Figures were prepared with *PyMOL* (http://www.pymol.org).

## 3. Results and discussion   

### 3.1. PA4991 encodes a flavin-containing protein   

**Figure 1:**
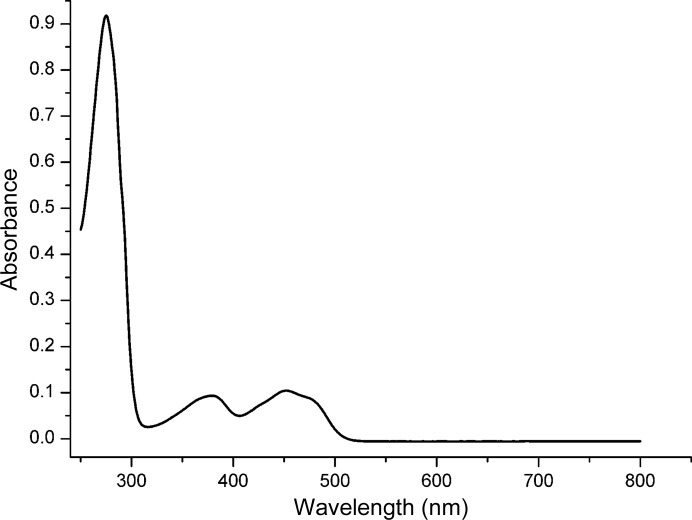
UV–visible absorption spectrum of recombinant PA4991 from *P. aeruginosa*. The spectrum shows the characteristic maxima for FAD-containing proteins at 365 and 445 nm. The sample consisted of 20 µ*M* PA4991 in in a solution of 25 m*M* Tris–HCl buffer pH 8.0, 0.15 *M* NaCl.

**Figure 2:**
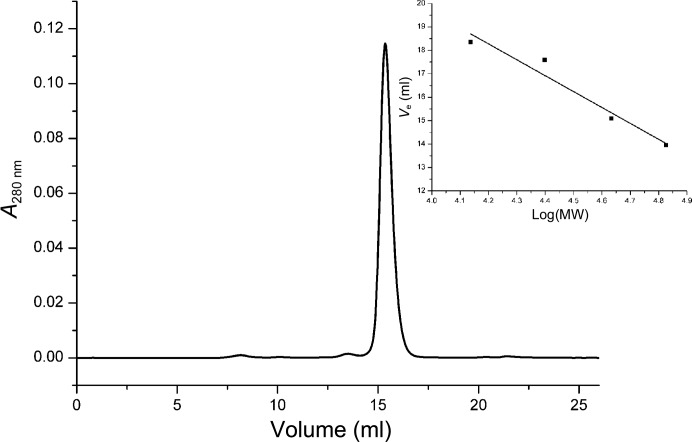
Elution profile of PA4991 from analytical size-exclusion chromatography using a Superdex 200 10/300 column (GE Healthcare) equilibrated with 25 m*M* Tris–HCl buffer pH 8.0 containing 150 m*M* NaCl. The column was calibrated with ribonuclease A (13.7 kDa), chymotrypsinogen A (25 kDa), ovalbumin (43 kDa), albumin (67 kDa) and blue dextran (2 MDa). The calibration curve is shown in the inset.

Locus PA4991 had been annotated to encode a flavin-binding protein of 391 amino acids (Winsor *et al.*, 2011 ▸). The construct used in this study resulted in soluble, folded protein of yellow colour that could be purified to homogeneity as judged from SDS gels. The UV–visible spectrum of PA4991 shows absorption maxima at 365 and 445 nm characteristic of flavin-binding proteins (Fig. 1 ▸). Analytical gel chromatography suggests that PA4991 is a monomer in solution. The protein elutes as a single peak corresponding to a molecular mass of 43.8 kDa when examined on an analytical size-exclusion chromatography column (Fig. 2 ▸), which is in good agreement with the mass of 42.2 kDa calculated from the amino-acid sequence.

### 3.2. Structure determination   

**Figure 3:**
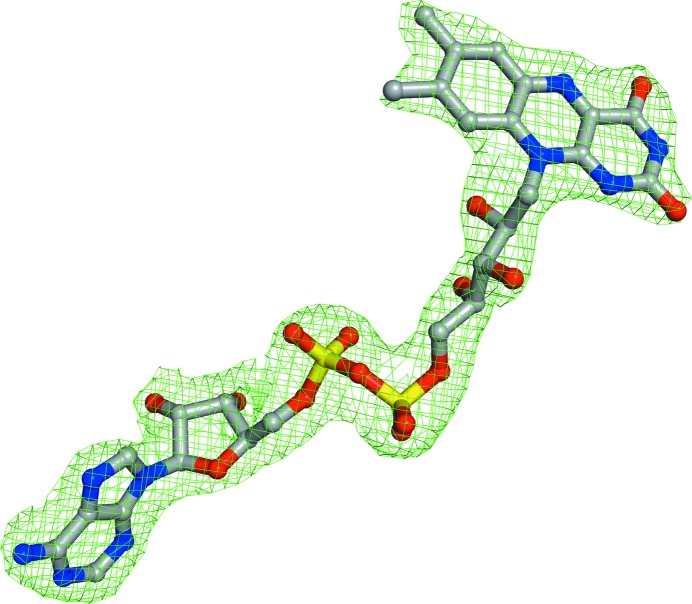
Simulated-annealing 2*F*_o_ − *F*_c_ OMIT electron-density map of the bound FAD molecule contoured at 1σ. The map was calculated using *PHENIX* (Adams *et al.*, 2010 ▸).

Attempts to determine the crystal structure of PA4991 using experimental phasing were not successful. We did not obtain diffracting crystals of the selenomethionine-substituted enzyme and soaking with heavy-metal salts did not result in useful derivatives for *ab initio* phase determination. We therefore resorted to molecular-replacement approaches, although the search models available in the PDB had a sequence identity of below 18%. Molecular-replacement runs with *Phaser-MR* (Bunkóczi *et al.*, 2013 ▸) using the coordinates of such putatively related structures did not produce interpretable electron-density maps. The structure of PA4991 was eventually solved by a combination of molecular replacement using a truncated search model generated from the sequence of PA4991 with *Rosetta* (Kim *et al.*, 2004 ▸) and phase improvement using a low-occupancy heavy-metal derivative obtained by soaking crystals with phenylmercuric chloride. The refined model of PA4991 consists of one polypeptide chain comprising residues 1–391, one FAD molecule, 41 water molecules and two Hg^2+^ ions bound on opposite sides of the thiol group of Cys245, albeit with low occupancy. Three loop regions, residues 55–67, 106–125 and 248–249, lacked electron density and appeared to be disordered in the crystal, and therefore were not modelled. Crystallographic refinement resulted in a model with statistics as expected for this resolution (Table 1 ▸). The 2*F*_o_ − *F*_c_ map for the bound cofactor FAD illustrates the quality of the electron-density maps in well ordered regions of the enzyme structure (Fig. 3 ▸).

### 3.3. Overall structure   

**Figure 4:**
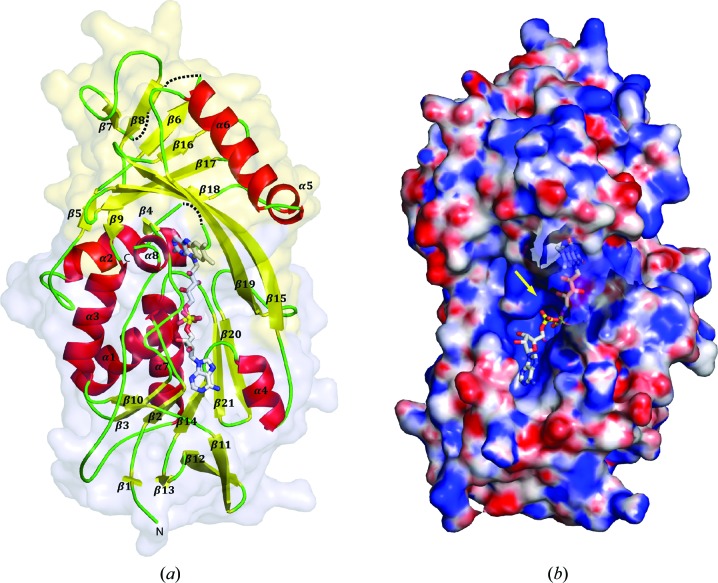
Overall structure of PA4991. (*a*) The surface of the FAD-binding domain is shown in light blue and that of the substrate-binding domain in light yellow. Secondary-structure elements are labelled and shown as a cartoon model with red α-helices and yellow β-strands. Disordered regions are displayed as dotted lines. The FAD molecule is shown in a stick representation. (*b*) Electrostatic potential surface of PA4991. The FAD-binding pocket and entrance to the active site is highlighted with a yellow arrow. The bound FAD molecule is shown as a stick model.

PA4991 consists of two domains: the canonical FAD-binding domain of the glutathione reductase family (Dym & Eisenberg, 2001 ▸) and a second domain that is most likely involved in substrate binding (Fig. 4 ▸). The α/β FAD domain consists of three chain segments: residues 1–95, 153–227 and 313–391. The domain is dominated by a central six-stranded β-sheet with the sixth strand antiparallel to the others. This sheet is flanked by helices α1 and α7 on one side and helix α4 on the other. This domain also contains the β-meander motif (β11, β12, β13) typical of this GR domain, but in PA4991 this sheet contains an additional short β-strand comprising residues 5–6, extending this structural feature to a four-stranded mixed β-sheet.

The second domain consists of peptide segments 96–152 and 223–312. The dominating feature of this domain is a mixed eight-stranded β-sheet with topology β7, β8, β6, β16, β17, β18, β15, β19 flanked by two helices, α5 and α6, on one side. Two of these strands, β15 and β19, extend from the FAD domain and are part of the binding site of the isoalloxazine ring of FAD.

### 3.4. Quaternary structure   

The asymmetric unit of the PA4991 crystals contains one monomer of the enzyme. An analysis of the crystal packing using *PISA* (Krissinel & Henrick, 2007 ▸) does not show any large protein–protein interfaces that would suggest a stable oligomeric structure. The largest interfaces of 460 and 540 Å^2^ are more typical of crystal-packing interactions than of protein–protein interfaces in oligomeric proteins (Ponstingl *et al.*, 2000 ▸). We conclude that PA4991 is a monomeric protein both in the crystal and in solution (Fig. 2 ▸).

### 3.5. Relation to other FAD oxidoreductases   

A search of the Protein Data Bank for structurally related proteins using *DALI* (Holm & Rosenström, 2010 ▸) returned a number of FAD-binding proteins, all with a very low degree of sequence identity to PA4991. The top hits were the β-subunit of the l-proline dehydrogenase complex from *Pyrococcus horikoshii* with an r.m.s.d. of 3.3 Å, 16% sequence identity and a *Z*-score of 26.6 (Tsuge *et al.*, 2005 ▸), *E. coli* glycerol-3-phosphate dehydrogenase with an r.m.s.d. of 3.4 Å, 14% sequence identity and a *Z*-score of 26.4 (Yeh *et al.*, 2008 ▸), glycine oxidase from *Bacillus subtilis* with an r.m.s.d. of 3.5 Å, 14% sequence identity and a *Z*-score of 26.2 (Settembre *et al.*, 2003 ▸; Mörtl *et al.*, 2004 ▸), the α-subunit of sarcosine oxidase from *Corynebacterium* sp. U-96 with an r.m.s.d. of 3.6 Å, 16% sequence identity and a *Z*-score of 26.1 (Moriguchi *et al.*, 2010 ▸) and α-glycerophosphate oxidase from *Streptococcus* sp. with an r.m.s.d. of 3.6 Å, 15% sequence identity and a *Z*-score of 25.3 (Colussi *et al.*, 2008 ▸). All of these proteins belong to the GR family of flavoenzymes and share the two-domain core with PA4991. However, glycerol-3-phosphate dehydrogenase and α-glycerophosphate oxidase contain additional domains that are not found in PA4991. The quaternary structures of these enzymes cover a wide range of assemblies from monomeric (glycerol-3-phosphate dehydrogenase) to hetero-octameric (l-proline dehydrogenase).

### 3.6. FAD-binding site   

FAD is bound noncovalently to PA4991 in an extended conformation in a manner similar to that observed in other members of the GR_2_ family (Dym & Eisenberg, 2001 ▸). Most of the interactions of the cofactor with the protein are through the FAD-binding domain, but the isoalloxazine ring is located at the domain interface and interacts with residues from both domains. The ADP moiety of FAD binds to the classic nucleotide-binding fold, including the diphosphate-binding fingerprint motif G*x*G*xx*G, which in PA4991 is represented by the sequence Gly13-Gly14-Gly15-Ile16-Ala17-Gly18 at the end of strand β2 in the central β-sheet of the domain. The diphosphate group points towards the N-terminus of helix α1 and the turn comprising residues 43–48.

**Figure 5:**
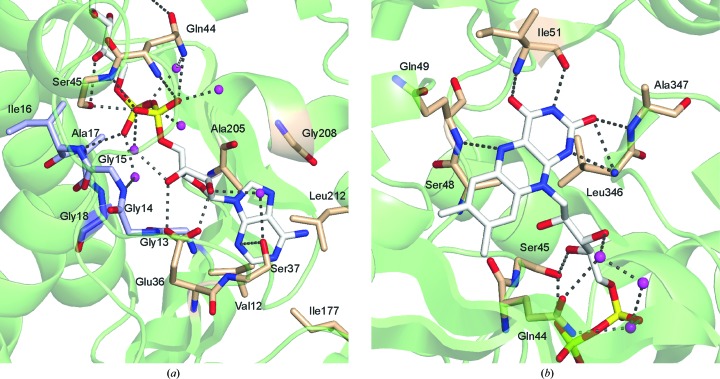
FAD–PA4991 interactions. PA4991 C, N and O atoms are coloured yellow, blue and red, respectively. Water molecules are shown as magenta spheres. Hydrogen bonds (3.2 Å cutoff) are shown as dotted lines. (*a*) Binding pocket of the adenosine moiety of FAD. The characteristic fingerprint motif Gly13-Gly14-Gly15-Ile16-Ala17-Gly18 is shown in blue. (*b*) Surroundings of the isoalloxazine ring of FAD in the active site of PA4991.

The adenine moiety is bound in a pocket lined by Val12, Ser37, Ile177, Ala205, Gly208 and Leu212 and is sandwiched between the side chain of Ser37 and Ala205 (Fig. 5 ▸ *a*). The C2 and C3 hydroxyl groups of the ribose ring form hydrogen bonds to a glutamic acid residue, Glu36, characteristic of this fold (Wierenga *et al.*, 1986 ▸). The P1 phosphate group is bound to the main-chain N atom of Ser45, the side chains of Ser45 and Gln44 and two ordered water molecules. The P2 phosphate group interacts with the main-chain N atom of Ala17 and three water molecules (Fig. 5 ▸ *a*). Van der Waals inter­actions dominate in the binding of the ribityl moiety, but a hydrogen bond from the C4′ hydroxyl group to the side chain of Ser45 also contributes to FAD recognition and binding.

The isoalloxazine ring is located in an accessible pocket with the *re* face of FAD facing the solvent. The electron-density maps do not indicate bending of the isoalloxazine ring as observed in the structure of α-glycerophosphate oxidase determined at a similar resolution (Colussi *et al.*, 2008 ▸), but are most consistent with a planar conformation of the aromatic ring system. The *si* face of the isoalloxazine ring is not accessible and packs against Ser48. The ring is anchored to the protein through a series of hydrogen bonds, all involving main-chain atoms of the polypeptide chain (Fig. 5 ▸ *b*). Hydrogen bonds are formed between the N1 atom of FAD and the main-chain N atom of Leu346, the N3 atom and the main-chain O atom of Ile51, the N5 atom and the main-chain N atom of Gln49, the O2 atom and the main-chain amides of Leu346 and Ala347, and the O4 atom and the main-chain N atom of Ile51. With the exception of the interaction of Glu36 with the ribose ring, there is little sequence conservation in residues involved in FAD binding in other members of this structural family, most likely because the majority of the interactions are through main-chain atoms.

### 3.7. Active site and enzyme assays   

On the *re* face of the isoalloxazine ring there is an open cleft that is accessible from the solvent. Accessibility of the cofactor in PA4991 appears in part to be controlled by the loop comprising residues 54–67, which is disordered in PA4991. Crystal structures of related enzymes with bound ligands, for instance glycerol-3-phosphate dehydrogenase with bound dihydroxyacetone phosphate (Yeh *et al.*, 2008 ▸), show that the corresponding loops fold over the active site and sequester the bound ligand. In the vicinity of the isoalloxazine ring there are the side chains of His53, Arg316 and Lys345 that potentially could be involved in ligand binding and/or catalysis. While in most of the distantly related structural homologues only one or two of these residues are structurally conserved, all three residues are found in the active site of glycerol-3-phosphate dehydrogenase. Arg316 is involved in the binding of the phosphate group of the substrate analogues glyceric acid 2-phosphate and glyceraldehyde-3-phosphate (Yeh *et al.*, 2008 ▸). The structurally equivalent Arg302 in glycine oxidase participates in the binding of the carboxyl group of the active-site ligands *N*-acetylglycine and glycolate, respectively (Settembre *et al.*, 2003 ▸; Mörtl *et al.*, 2004 ▸).

The conservation of some of the active-site residues of glycerol-3-phosphate dehydrogenase and glycine oxidase in PA4991 suggested that the enzyme might show corresponding enzymatic activities. However, enzyme assays using glycerol-3-phosphate with NAD^+^ or NADP^+^ as co-substrate and dihydroxyacetone phosphate as substrate and NADH or NADPH as co-substrates, respectively, did not show any enzymatic activity. We also tested 41 putative amines as potential substrates (including the 20 proteinogenic amino acids, d-amino acids, unconventional and modified amino acids and biogenic amines). No enzymatic activity could be detected in these cases either, suggesting that PA4991 shows a very different substrate specificity compared with its closest structural homologues.

## 4. Conclusions   

In the course of the structure determination of PA4991, phasing using heavy-metal derivatives or selenomethionine substitution failed. Initially, molecular replacement also did not provide a solution owing to a lack of suitable templates in the Protein Data Bank. However, a combination of molecular replacement using a truncated model produced by the *Rosetta* software and a weak heavy-metal derivative provided sufficient phase information for structure determination to succeed. This approach might also be applicable in other cases where phase information is lacking.

Analysis of the structure of PA4991 identifies this enzyme as a member of the GR_2_ family of flavoenzymes. Enzymatic assays did not support a function as a G3P dehydrogenase/oxidase or an amino-acid oxidase as hypothesized based on the three-dimensional structure. The conservation of the active-site arginine residue involved in the binding of negatively charged groups of the substrate in these enzymes suggests that the unknown substrate of PA4991 might also carry a negatively charged phosphate or carboxyl group. The function of this essential enzyme in the virulence and survival of *P. aeruginosa* in the host nevertheless remains elusive.

## Supplementary Material

PDB reference: FAD-dependent oxidoreductase PA4991, 5ez7

## Figures and Tables

**Table 1:** Data-collection and refinement statistics for PA4991 Values in parentheses are for the outer shell.

Data collection	
Space group	*P*2_1_
Unit-cell parameters (Å, °)	*a* = 37.9, *b* = 79.8, *c* = 63.2, β = 104.4
Wavelength (Å)	1.0093
Resolution (Å)	48.6–2.40 (2.53–2.40)
*R*_merge_	0.072 (0.298)
*R*_meas_	0.084 (0.345)
*R*_p.i.m._	0.042 (0.180)
Total No. of observations	106569
No. of unique reflections	14372
〈*I*/σ(*I*)〉	17.3 (5.3)
Completeness (%)	99.8 (98.7)
Multiplicity	7.4 (7.2)
Wilson *B* factor (Å^2^)	41.2
Refinement	
Resolution (Å)	48.6–2.40
No. of reflections	13625
*R*_work_/*R*_free_	0.173/0.228
No. of protein residues	391
No. of FAD molecules	1
No. of atoms	
Protein	2780
FAD	53
Hg^2+^ (0.35 occupancy)	2
Water molecules	41
*B* factors (Å^2^)	
Overall	40.0
Protein	41.3
FAD	25.1
Hg^2+^ (0.35 occupancy)	50.9
Water	31.1
R.m.s deviations	
Bond lengths (Å)	0.014
Bond angles (°)	1.72
Ramachandran plot (%)	
Favoured	96.4
Allowed	3.6
